# *Aelurostrongylus abstrusus* Infections in Domestic Cats (*Felis silvestris catus*) from Antioquia, Colombia

**DOI:** 10.3390/pathogens10030337

**Published:** 2021-03-13

**Authors:** Sara Lopez-Osorio, Jeffer Leonardo Navarro-Ruiz, Astrid Rave, Anja Taubert, Carlos Hermosilla, Jenny J. Chaparro-Gutierrez

**Affiliations:** 1 Grupo de Investigación CIBAV, Universidad de Antioquia UdeA, Medellín 050034, Colombia; sara.lopezo@udea.edu.co (S.L.-O.); jeffer.navarro@udea.edu.co (J.L.N.-R.); astridrave95@gmail.com (A.R.); 2 Institute of Parasitology, Justus Liebig University Giessen, 35392 Giessen, Germany; anja.taubert@vetmed.uni-giessen.de (A.T.); Carlos.R.Hermosilla@vetmed.uni-giessen.de (C.H.)

**Keywords:** *Aelurostrongylus abstrusus*, lungworms, Antioquia, Colombia

## Abstract

Although *Aelurostrongylus abstrusus* infections in domestic cats (*Felis silvestris catus*) have sporadically been reported in Colombia, there is still no data available on epidemiology nor on the biology of this neglected lungworm parasite. Thus, this epidemiological study aimed to evaluate the occurrence of patent *A. abstrusus* infections in domestic cats from the Colombian Federal State of Antioquia. In total, 473 fecal samples of indoor/outdoor domestic cats were collected and analyzed thereafter by the Baermann funnel migration technique for the presence of *A. abstrusus* first stage larvae 1 (L1). The occurrence of *A. abstrusus* was confirmed in 0.4% (2/473) of investigated cats. Due to the presence of patent *A. abstrusus* infections in investigated cats, it is unfailing to include this lungworm within differential diagnoses of feline pulmonary disorders. Despite the fact that the Baermann funnel technique is currently the cheapest and the gold standard diagnostic tool for feline aelurostrongylosis, this technique is still unknown by Colombian veterinary surgeons and rarely utilized in small animal veterinary clinics. The current survey intends to generate awareness on this neglected parasitosis and to be considered as a baseline study for future surveys monitoring feline aelurostrongylosis not only in domestic/stray cats but also in endemic wild felid species of Colombia.

## 1. Introduction

In Colombian territories, there is still no updated data on the prevalence of lungworms affecting domestic cats (*Felis silvestris catus*). The gastropod-borne parasite *Aelurostrongylus abstrusus* affects the respiratory tract of domestic as well as wild felids [1,2]. This lungworm species shares a similar life cycle to other closely related metastrongyloid parasites such as *Troglostrongylus brevior* and *Crenosoma vulpis* [3,4]. In the lungs, fertilized *A. abstrusus* females lay embryonated eggs from which first-stage larvae (L1) hatch and then ascend the respiratory tract up to the pharynx. Then, the L1 are swallowed and leave the definitive host via the feces [4,5]. Exogenous L1 of *A. abstrusus* can survive in the environment for up to 60 days until they infect terrestrial snails and slugs (i.e., *Arion lusitanicus, Limax maximus, Lissachatina fulica* [3,6], *Helix aspersa* [7], *Massylaea vermiculata* and *Helix lucorum* [8] and *Arion vulgaris* [9]), which represent the obligate intermediate hosts [1]. In gastropods, L1 develops into second-(L2) and third-(L3) larval stage in approximately 2 weeks. Feline hosts ingest either L3 infected-gastropods or paratenic hosts (e.g., rodents, reptiles, birds) carrying L3, and the life cycle is completed [5].

In cats, aelurostrongylosis manifestations can be subclinical or clinical, depending on infection dose and host immune responses [10]. Clinical symptoms include respiratory signs such as dyspnoea, abdominal breathing, coughing, sneezing and mucopurulent nasal discharge [10,11]. *Aelurostrongylus abstrusus* infections have been reported in several parts of the world; recently in Greece [12] and Italy [13] and also detected in other countries of Europe (Austria, Belgium, Bulgaria, France, Hungary, Portugal, Romania, Spain, Switzerland, and the United Kingdom) [14], in the USA [15,16], the Eastern Caribbean [17], and also in South American wildlife felid species, including jaguarundis (*Puma yagouaroundi*), margay (*Leopardus wiedii*) [18], and oncilla (*Leopardus tigrinus*) [19]. All these wild felid species are endemic within diverse regions of Colombia together with the presence of adequate intermediate hosts, which may contribute to *A. abstrusus* sylvatic life-cycle [3,20]. Nevertheless, the presence of the parasite has never been confirmed with molecular methods in the mentioned wild species.

As stated before, available data on the occurrence of *A. abstrusus* infections in domestic cats from Colombia remains poor and infections are mainly detected post mortem [21]. In 2003, Salamanca et al. [22] reported and treated a case of *A. abstrusus* in a domestic cat from Bogotá, Colombia. The presented animal was an indoor-outdoor cat living exclusively in the city, which emphasizes the presence of *A. abstrusus* not only in rural areas but also in urban areas [23]. The best diagnostic tool for detection of a patent *A. abstrusus*-infection in cats is the Baermann funnel migration technique, which detects shed L1 in feces but this technique, although being simple and cheap, is rarely utilized in Colombian small animals veterinary clinics for the diagnosis of feline aelurostrongylosis [2].

Although *A. abstrusus* infections have previously been reported to occur in domestic and wild felids in other South American countries, still few large-scale epidemiological surveys have been conducted on this lungworm in the last years [2,3]. Therefore, this study aimed to close this knowledge gap and further evaluate the occurrence of *A. abstrusus* in a large domestic cat population from Antioquia, Colombia.

## 2. Results

### Occurrence of Patent A. abstrusus

**Figure 1:**
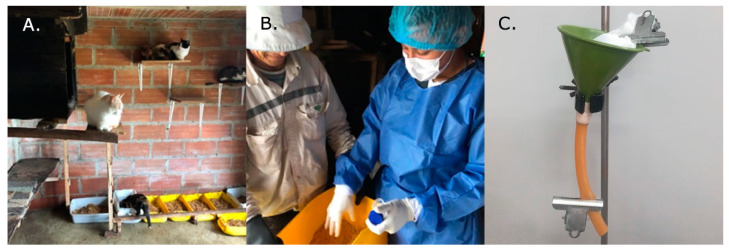
(**A**) Cat conditions in one of the animal shelters sampled. (**B**) Collection of the scat samples from a litter box. (**C**) Apparatus of a Baermann funnel for lungworm diagnosis.

We report here on the occurrence of patent *A. abstrusus* infections in a large domestic cat population (*n* = 473) of Antioquia. The cats included in this survey were indoor-outdoor domestic shorthair cats, between 2 months to 15 years of age (8.5 years average). The cats were fed with commercial dry cat food and sometimes with human food residue. The presence of slugs was reported in 2 of 5 shelters, all owners reported occasional respiratory signs during the rainy season (April–May). All of the cats were vaccinated (anti-rabies vaccine) and dewormed three months before the sampling. Detection of vital and motile *A. abstrusus* L1 in fecal material (2/473) through various Baermann funnel apparatuses and light microscopy analysis was achieved with ease (Figure 1).

Overall, the occurrence of *A. abstrusus* was rather low (0.4%) in these animal shelters as only two cats (2/473) resulted positive for *A. abstrusus* L1. The magnitude of larval shedding in these two *A. abstrusus*-infected cats were also quite low with an average of one larva per power vision field (20× magnification). Infected cats did not show any clinical signs of respiratory disease.

Samples analyzed were collected from animal shelters and step homes. The population of cats in each shelter was between 5 to 318, females and males, aged from 2 months to 15 years. Cats were isolated in specific groups of age, in cement floor buildings, with litter boxes. Cats had apparently no contact with prey animals such as birds, mice, and toads but this cannot be completely ruled out. Apparently, terrestrial gastropods (snails and slugs) were not reported to occur within the premises but again it cannot be ruled out that some gastropods may have entered these shelters in the past. All animals analyzed were neutered, without respiratory symptoms, and periodic vaccination and anthelminthic treatments were carried out.

**Figure 2:**
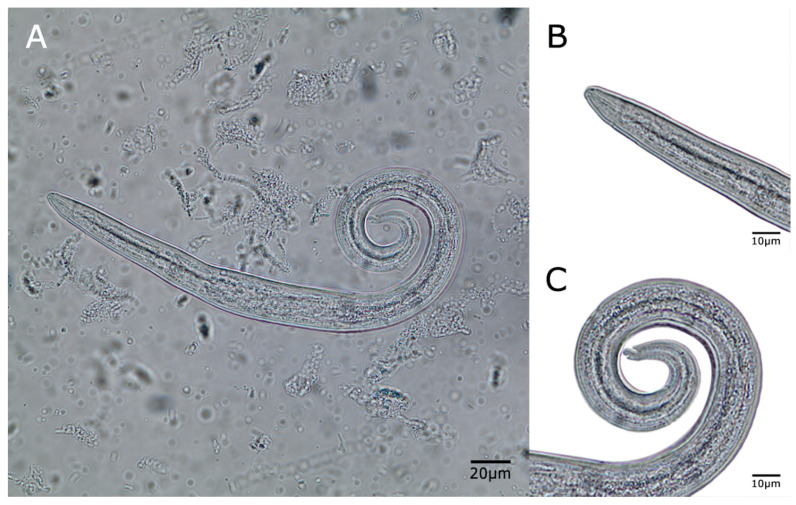
(**A**) *Aelurostrongylus abstrusus* larvae 1 (L1). Details of (**B**) anterior part of larvae and (**C**) larval tail with characteristic notched and S-shaped tip. 400× magnification. Scale bar = 20 µm (**A**), 10 µm (**B**,**C**).

Although all feces were collected from cat litter boxes (Figure 1), in some examined samples non-parasitic free-living earth nematodes were also detected (data not shown). Collected *A. abstrusus* L1 showed classical anterior and posterior morphological features (round head, short and terminal oral opening; tail with S-shape, with a dorsal kink, distinct deep dorsal and ventral incisures, and a terminal knob-like extremity [24,25] (Figure 2) and morphometric characteristic (mean length 405.50 ± 0.3; mean width 17.20 ± 0.1).

## 3. Discussion

*A. abstrusus* is the most common nematode affecting the respiratory system of domestic cats. It can also infect wild felids under some epizootiological circumstances [19]. The Baermann technique is the routinely used diagnostic method for the identification of L1 in the feces [25]. However, the sensitivity of the method could be impaired by the intermittent shedding of the larvae [26,27], the prepotency period, and minimal larval excretion in subclinical infections [25].

*A. abstrusus* represents one of the most important parasitic nematode species causing respiratory diseases in domestic and wildlife cats worldwide [23,28]. In fact, infections with this parasite are widespread in North-, South America, and Europe [19,24]. Consistently, in South America, the presence of *A. abstrusus* has previously been reported sporadically as case reports but few large epidemiological studies have been conducted in domestic felids so far [2,3]. Some of these studies have been performed in Brazil [29,30,31,32,33,34], Argentina [35,36,37], Uruguay [38,39], Chile [40], and Colombia [41]. In the Colombian survey, Echeverry et al. [41] also found a rather low *A. abstrusus* prevalence of 0.21% (1/121) in indoor cats from Quindío, which is similar to our findings. Nevertheless, that study used the Ritchie technique, which is a sedimentation technique considered a less accurate diagnostic tool than the Baermann funnel technique for detection of metastrongyloid L1 stages [2]. In fact, the Ritchie technique only provides positive results in cases of heavily parasitized animals with high larval shedding [22]. The use of this technique might have led to an underestimation of the real prevalence of *A. abstrusus*. Results obtained by Traversa et al. [25] demonstrated the importance of the specificity of a diagnostic technique being used for the detection of *A. abstrusus* positive animals.

In addition, it is important to consider that the annual deworming with fenbendazole/praziquantel that the cats examined in the present study received is not expected to be adequate to prevent *A. abstrusus* infection. Fenbendazole is effective in treating aelurostrongylosis if administered at 50 mg/kg of body weight Fenbendazole treatment may require way more than three days of administrations [42]. A spot-on formulation containing emodepside and praziquantel showed high efficacy against *A. abstrusus* infection in field conditions [43,44] as well as other spot-on formulations containing either eprinomectin or moxidectin [45]. Additionally, another effective oral combination was milbemycin oxime and praziquantel administered at 4 mg/kg and 10 mg/kg respectively, three times as a single dose with two-week intervals [46]. Another therapy that was found to be effective and safe in the treatment of aelurostrongylus in cats was a formulation that contained imidacloprid 10% and moxidectin 1% [43]. In addition, the effectiveness of an off-label spot-on combination has been demonstrated [47].

No further parasitic feline metastrongyloid L1, i.e., *T. brevior, G. paralysans,* and *Angiostrongylus chaubadi*, were detected in this Colombian epidemiological survey, although closely related metastrongyloid *T. brevior* has previously been reported to occur in Colombia detected in terrestrial gastropod intermediate hosts [3]. *T. brevior* is a parasite usually affiliated to wild felids [8] and further studies are needed to determine its occurrence in wild felids from South America. The first-stage larvae of *A. abstrusus* are identified based on their length and on the morphological characteristics of the anterior and posterior ends [28]. Most descriptions report that L1s of *A. abstrusus* are ~360–415 μm long [48] with a rounded head with a terminal oral opening and a kinked (S-shaped) tail with distinct knob-like or small finger-like projections at the tip with cuticular spines [48]. First stage larvae of *A. abstrusus*, *T. brevior,* and *T. subcrenatus* are rather similar, except for their slightly different body lengths and the knob-like terminal end of *A. abstrusus.* [49].

The Baermann technique has been demonstrated to be the most sensitive test (81.8%) for the detection of *A. abstrusus* infection in the lungs [50]. Other authors have found that flotation methods (i.e., McMaster and flotation with saline saturated solution) are not adequate for the diagnosis of this lungworm parasite. Indeed, the lack of reports on pulmonary nematodes in cats in Colombia might be explained by the fact that the Baermann funnel technique is still rarely performed as a routine veterinary diagnosis [3,20]. Generally, cat feces are analyzed in most cases with saturated solution techniques or sedimentation techniques for parasite detection. These techniques are inappropriate for feline lungworm diagnosis, unleashing a chronic infection, or even premature death of heavily *A. abstrusus* parasitized cats. This study may be limited because of the Baermann technique and its diagnostic performance and sensitivity can be compromised by various factors: the inability to isolate larvae in the pre-patent period, irregularity shedding of the larvae, and cessation of shedding larvae in some infected cats, which could lead to false-negative results [26,28]. To increase the accuracy of detection of *A. abstrusus* in future studies, Baermann’s technique should be performed at least on three consecutive days to improve the sampling process to assure that all cats are included. Therefore, more epidemiological surveys on lungworm infections in domestic felids as well as their intermediate/paratenic-hosts are still required in Colombia [3,20]. Furthermore, novel diagnostic tools, such as serological and molecular approaches [12,51,52,53,54], together with the Baermann technique, should be used for future large-scale national epidemiological surveys on feline lungworm infections, not only in domestic but also in wildlife animals of South America.

## 4. Materials and Methods

Cat fecal samples were collected in the Aburrá Valley, Antioquia, Colombia from August to December 2019. The cats were from animal shelters or step homes. All of the examined animals included in this survey were indoor-outdoor domestic shorthair cats, neutered females and males between 2 months to 15 years of age. No cats showed clinical signs of respiratory diseases during the sampling, although coughing and diarrhea were reported by the owners. The cats were dewormed yearly with an oral combination of fenbendazole-praziquantel

Feces were collected from sandboxes and labeled with numbers before the coprological examination. The owners of the shelters cleaned the boxes in the night (9 p.m.) of the day before and put in clean litter. Samples were collected in the morning of the next day. The cats were observed during the sampling period (approximately 3 h each morning) by veterinary practitioners associated with the study, for respiratory signs. Additionally, the owners and helpers that lived with the cats observed the cats daily for the presence of potential clinical signs. Collected cat samples were transferred immediately to the Veterinary Parasitology Laboratory of the Agrarian Sciences Faculty, Medellin, University of Antioquia. Samples were examined by the Baermann funnel migration technique for the presence of larvae [24,55]. Obtained *A. abstrusus* L1 were morphologically identified according to previous reports [55]. Only L1 with a characteristic notched and S-shaped tip at the posterior end were determined as *A. abstrusus* with small finger-like or distinct knob-like projections at the tip of cuticular spines [5,11,24,25].

## 5. Conclusions

The presence of *A. abstrusus* in domestic cats, as well as natural intermediate hosts, is a fact [13], and thus it should be included in the differential diagnoses of feline pulmonary disorders in Colombia. The Baermann funnel technique is the most viable and cheapest diagnostic tool for this neglected pulmonary parasitosis [17]. It is very important to include this technique in routine coproparasitological diagnosis by veterinary surgeons dedicated to small animals and wildlife conservation within Colombian territories. Since the correct diagnosis of *A. abstrusus* will contribute to improved health of affected domestic and wild felids and result in proper anthelminthic treatments impeding further aelurostrongylosis spreading.

## Data Availability

No new data were created or analyzed in this study. Data sharing is not applicable to this article.
